# Circulating Tumour DNA Is a Biomarker of Response in Angioimmunoblastic T-Cell Lymphoma

**DOI:** 10.3390/ijms26146719

**Published:** 2025-07-13

**Authors:** Costas Kleanthes Yannakou, Simon Wu, Karthik Rajah, Chathuri Abeyakoon, Caitlyn Nguyen-Ngo, Yan Zhuang Yap, James Sheldon, Piers Blombery, Henry Miles Prince

**Affiliations:** 1 Epworth HealthCare, Melbourne 3002, Australia; 2 Department of Clinical Pathology, The University of Melbourne, Melbourne 3010, Australia; 3 Sir Peter MacCallum Department of Oncology, The University of Melbourne, Melbourne 3010, Australia; 4 Peter MacCallum Cancer Centre, Melbourne 3000, Australia; 5 Dorevitch Pathology, Melbourne 3084, Australia; 6 QML Pathology, Brisbane 4172, Australia; 7 Monash Health, Melbourne 3168, Australia

**Keywords:** angioimmunoblastic T-cell lymphoma, circulating tumour DNA, measurable residual disease

## Abstract

Angioimmunoblastic T-cell lymphoma (AITL) is a rare and aggressive subtype of non-Hodgkin lymphoma, the monitoring of which is largely restricted to radiological methods. Diagnosis relies on identifying characteristic clinicopathological features, supported by the detection of recurrent somatic mutations in *RHOA*, *TET2*, *IDH2* and *DNMT3A*. The characteristic molecular profile of AITL and the high levels of circulating tumour DNA (ctDNA) measurable in AITL before treatment makes this an attractive lymphoma subtype in which to further investigate the role of ctDNA monitoring. The detection of somatic mutations in pre-treatment AITL-containing tissue samples was compared to those detected in pre-treatment ctDNA samples in a cohort of 12 patients. Changes in ctDNA somatic mutation burden over time were then correlated with radiological response. All six paired pre-treatment ctDNA and tissue samples had variants in common. All (8/8) previously ctDNA-detectable *IDH2* and *RHOA* variants were undetectable in ctDNA samples at the time of end-of-treatment complete metabolic response (CMR). In comparison, the majority of both previously ctDNA-detectable *DNMT3A* variants (3/4) and *TET2* variants (6/11) were detectable in ctDNA samples at the time of end-of-treatment CMR. These observations suggest that *IDH2*/*RHOA* variants may be more reliable markers of measurable residual disease in AITL than *DNMT3A*/*TET2* variants.

## 1. Introduction

Angioimmunoblastic T-cell lymphoma (AITL), a malignancy of the T follicular helper cell, is a rare and aggressive subtype of non-Hodgkin lymphoma accounting for approximately 30% of peripheral T-cell lymphomas [1,2]. Establishing the diagnosis of AITL is often challenging given its varied clinical presentations that include autoimmune manifestations. At present, diagnosis relies on identifying characteristic clinicopathological features, supported by the detection of recurrent somatic mutations. Peripheral T-cell lymphoma of follicular helper cell phenotype (PTCL-TFH) is a related malignancy of the T follicular helper cell that shares similarly recurrently mutated genes with AITL. Despite a complete remission rate of approximately 60% to first-line CHOP-based (cyclophosphamide, doxorubicin, vincristine and prednisolone) treatment, long-term outcomes with chemotherapy alone are poor in AITL, with a 5-year progression-free survival and overall survival of 32% and 44%, respectively [3].

Currently, disease monitoring in AITL is largely restricted to radiological methods, limiting the assessment of disease evolution over time. While the role of combination ^18^F-fluorodeoxyglucose positron emission tomography and computed tomography (PET/CT) is widely accepted in AITL staging and end-of-treatment response assessment, the role of interim PET/CT is less clear [4,5,6].

The measurement of circulating tumour DNA (ctDNA) has been evaluated for both accurate diagnosis and monitoring of measurable residual disease (MRD) in various malignancies. The advantages of such testing being the relative non-invasiveness of peripheral blood sampling, the ability to assess spatial heterogeneity and ongoing improvements in the rapidity, accuracy and reproducibility of results [7].

Amongst haematological malignancies, the evidence for the value of ctDNA assessment is most robust for diffuse large B-cell lymphoma (DLBCL) where ctDNA is detectable in up to 98% of cases. Moreover, changes in the amount of ctDNA after treatment have been evaluated as a potential biomarker [8]. The measurement of ctDNA in classical Hodgkin lymphoma has also been correlated with PET/CT response [9].

The role of ctDNA in T-cell lymphomas is less well-defined. However, the characteristic molecular profile of AITL and the high levels of ctDNA measurable in AITL before treatment makes this an attractive lymphoma subtype in which to further investigate the role of ctDNA monitoring [10]. Four genes are recurrently mutated in 80% of cases of AITL: *RHOA* (50–70% of cases), *TET2* (47–83%), *IDH2* (20–45%) and *DNMT3A* (20–30%) [11,12,13,14,15,16]. Almost all *RHOA* mutations detected in AITL are *RHOA* G17V. *IDH2* mutations appear to be highly recurrent in AITL but not in other T-cell lymphomas [17,18,19]. Of note, *TET2*, *IDH2* and *DNMT3A* mutations are recurrent in myeloid malignancies, while *TET2* and *DNMT3A* mutations are described in clonal haematopoiesis of indeterminate potential [20,21,22].

Multiple studies have demonstrated concordance between the detection of *RHOA* G17V mutations in both ctDNA and tumour AITL samples [10,16,23]. There is also promising data regarding ctDNA as a potential biomarker of response in AITL [10,16,24]. Serial sampling has demonstrated changes in *RHOA* mutation burden after treatment and the emergence of new clones at the time of relapse [10]. There is also emerging data on the multi-step tumourigenesis of AITL, where *TET2* and *DNMT3A* mutations have been found in multiple morphologically normal cell lineages as well as in the tumour tissue of AITL patients, suggesting that AITL may originate from *TET2* and *DNMT3A* mutated premalignant clones [25,26,27]. In contrast, *RHOA* and *IDH2* mutations are found almost exclusively in AITL cells, indicating that these mutations are acquired later in AITL development [25,27,28,29,30,31]. Given the above, we hypothesised that *RHOA* and *IDH2* mutations may be suitable markers of MRD in AITL and sought to study their performance as such in comparison to radiological response in patients undergoing treatment.

The aims of this study were to describe the somatic mutations detected in pre-treatment AITL-containing tissue samples, compare the detection of somatic mutations in pre-treatment AITL-containing tissue samples to those detected in pre-treatment ctDNA samples, and to correlate the changes in ctDNA somatic mutation burden over time with radiological response.

## 2. Results

Eight females and four males were enrolled in this study with a median age at diagnosis of 69.5 years (range 51–77) and a median follow-up of 19.5 months (range 6–50). The pre-treatment Lugano staging, treatment regimens and end-of-treatment PET/CT responses are described in Table 1. Eleven patients received first-line treatment while one patient (patient 5) was treated for relapse.

Five patients progressed or relapsed (patients 2, 4, 5, 6 and 7). Patient 5 was enrolled at the time of first relapse, then subsequently developed DLBCL. Patient 3 developed acute myeloid leukaemia post cytotoxic therapy (AML-pCT). Six patients maintained a complete metabolic response (CMR) until the time of the last follow-up (patients 1 and 8–12).

Genomic sequencing data was available from 28 ctDNA samples tested over the study period, including 6 paired with pre-treatment tissue samples. Ten samples were collected during CMR, six samples were collected during surveillance, four samples were collected after progression/relapse and two samples were collected after the diagnosis of another haematological malignancy (patient 3 developed AML-pCT, and patient 5 developed DLBCL).

Somatic variants were identified in all twelve pre-treatment tumour samples. Eleven samples contained at least two variants in genes known to be recurrently mutated in AITL. The mutation frequencies for genes recurrently mutated in AITL were *DNMT3A* = 8/12 (66.7%, 95% confidence interval (CI) = 34.9–90.1%)*, IDH2* = 8/12 (66.7%, 95% CI = 34.9–90.1%)*, RHOA* = 10/12 (83.3%, 95% CI = 51.6–97.9%) and *TET2* = 11/12 (91.7%, 95% CI = 61.5–99.8%) (see Table 2 and Supplementary Table S1). *RHOA* variants consistently involved the G17 amino acid position, with G17V detected in nine cases and one case with G17E detected in conjunction with S26R in patient 12. Similarly, all eight cases with an *IDH2* mutation involved the R172 amino acid position.

All six paired pre-treatment ctDNA and tissue samples had variants in common. In four cases, either an *IDH2* or *RHOA* variant was detectable in both samples, while all six samples had detectable variants in *DNMT3A* = 5/6 patients (83.3%, 95% CI = 35.9–99.6%) or *TET2* = 6/6 patients (100.0%, 95% CI = 54.1–100%).

Serial monitoring of ctDNA identified three main findings. Firstly, all eight (100.0%, 95% CI = 63.1–100%) previously ctDNA-detectable *IDH2* and *RHOA* variants were undetectable in ctDNA samples at the time of end-of-treatment CMR. During subsequent surveillance, a ctDNA-detectable *IDH2* variant at 1.3% VAF and a ctDNA-detectable RHOA variant at 1.4% VAF were detected in patient 4 and patient 7, respectively, both of whom subsequently relapsed.

Secondly, the majority of both previously ctDNA-detectable *DNMT3A* variants = 3/4 (75.0%, 95% CI = 19.4–99.4%) and *TET2* variants = 6/11 (54.5%, 95% CI = 23.4–83.3%) were detectable in ctDNA samples at the time of end-of-treatment CMR.

Lastly, new variants developed in the ctDNA over time in patients during surveillance or at the time of relapse. Additional *DNMT3A* and *TET2* variants developed in one (patient 1) and two patients (patients 1 and 3), respectively. A *KRAS* variant was detected in patient 3 after they developed AML-pCT. *TP53* variants developed in four patients, two of whom relapsed (patients 2 and 4). A *RUNX1* variant developed in patient 11, who remained in remission at the time of last follow-up.

Three patients (patients 2, 4 and 6) developed biopsy-confirmed relapsed AITL after attaining a CMR. Notably, an *IDH2* or *RHOA* variant detected in the initial pre-treatment tumour sample was detectable in subsequent ctDNA monitoring in all of these patients following relapse. These variants were not detected in the ctDNA samples of patients 2 and 4 collected at the time of attaining a CMR—no corresponding remission ctDNA sample was available for patient 6. Of note, the *NOTCH1* variant detected at diagnosis in patient 2 was not detected at relapse.

**Figure 1:**
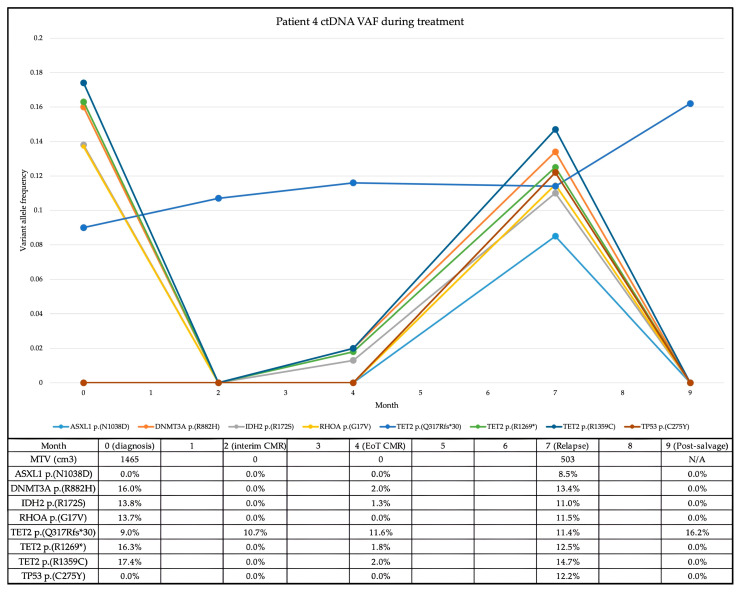
ctDNA variant allele frequencies during treatment and surveillance for patient 4. ctDNA: circulating tumour DNA; VAF: variant allele frequency; CMR: complete metabolic response; EoT: end of treatment; MTV: metabolic tumour volume.

In the case of patient 4, the re-emergence of the previously detected and subsequently cleared *IDH2* variant within the ctDNA preceded radiological relapse by three months (see Figure 1). End-of-treatment PET/CT confirmed relapse, in conjunction with the re-emergence of all the previously detected variants as well as a newly acquired *TP53* variant.

Two patients developed additional haematological malignancies during the study period. Patient 3 developed AML-pCT after receiving treatment for AITL and in the context of persistent ctDNA-detectable *DNMT3A* and *TET2* variants during CMR. The *TET2* variant was also observed in the bone marrow aspirate at the time of AML-pCT diagnosis, and both variants were detected in a surveillance ctDNA sample 12 months after AML-pCT diagnosis, with additional variants in *TET2* and *KRAS* (Supplementary Table S1).

In the case of patient 5, enrolled at the time of relapse, neither *IDH2* nor *RHOA* variants were detected in the pre-treatment samples—only *DNMT3A* and *TET2* variants were detected. The patient did not attain a CMR and these variants persisted in the ctDNA after treatment for AITL. The patient subsequently developed DLBCL which harboured the same *DNMT3A* and *TET2* variants as the prior AITL—these were also detectable in the ctDNA (Supplementary Table S1).

## 3. Discussion

This study assessed the representation of somatic mutations in the ctDNA of AITL patients and described the changes in these mutations in response to treatment. ctDNA quantification did correlate with radiological response in patients with AITL.

Mutations in *DNMT3A* and *TET2* were detected in the majority of patients with AITL in both pre-treatment tumours (8/12 and 11/12, respectively) and in ctDNA where available (5/6 and 6/6, respectively). These mutations persisted in ctDNA at the time of CMR, including in patients who maintained a CMR, suggesting their presence within pre-lymphoma clones and hence limiting their utility as markers of MRD in AITL [32,33]. The persistence of these mutations in the ctDNA at the time of CMR may predispose to the development of additional haematological malignancies, as occurred in one patient who developed AML-pCT [32]. A small proportion of previously ctDNA-detectable *DNMT3A* and *TET2* variants (6/15) became undetectable in ctDNA samples at the time of CMR—such variants are therefore likely found exclusively in AITL cells and may be considered potential markers of MRD.

In comparison, the persistence or re-emergence of *RHOA* and/or *IDH2* variants in the ctDNA consistently heralded relapse. Neither case in which *RHOA* (patient 7) or *IDH2* (patient 4) variants persisted in the ctDNA after treatment attained a lasting CMR, in keeping with their likely presence within AITL cells. In addition, there were no cases in which known *RHOA* and/or *IDH2* variants were not detectable in the ctDNA at the time of AITL relapse. In contrast, variants in genes not known to be recurrently mutated in AITL may not persist or re-emerge at the time of relapse, as demonstrated by the *NOTCH1* variant in patient 2.

Interestingly, two patients developed additional haematological malignancies—patient 3 developed AML-pCT and patient 5 developed DLBCL. In both cases, variants that had been detected in the pre-treatment AITL-containing tissue and ctDNA samples were also detected in the AML-pCT-containing and DLBCL-containing samples, respectively, as well as in subsequent ctDNA samples. The development of such additional haematological malignancies in the setting of AITL and the implication of common founder clones in the tumourigenesis of such cases has been previously described [32,33,34,35].

This cohort of 12 patients is comparable in size to other studies assessing ctDNA in AITL [10,23]. *RHOA* mutations in ctDNA have been reported in 18/40 patients (45%) diagnosed with T follicular helper cell lymphomas by Kim et al. and 6/9 patients (67%) by Sakata-Yanagimoto et al., while *IDH2* mutations were detected in ctDNA in 9/40 (23%) and 1/9 (11%) patients, respectively [10,23].

This exploratory study is limited by its retrospective design and modest size. Although the results lack the statistical significance required to form definitive conclusions or to assist in powering future prospective studies, they do nonetheless support further investigation of the role of ctDNA monitoring in AITL. Although this would be challenging given the rarity of AITL, it would allow for the robust validation of ctDNA as a biomarker of response as proposed by Kim et al. [10]. Radiological monitoring remains the standard approach in AITL; however, this study has demonstrated that ctDNA quantification can detect AITL relapse before PET/CT, and so may reasonably be considered an adjunctive disease-monitoring strategy.

## 4. Materials and Methods

We performed a retrospective, exploratory cohort study of patients diagnosed with AITL or PTCL-TFH between 2017 and 2023 at Epworth HealthCare (Melbourne, Australia) and Peter MacCallum Cancer Centre (Melbourne, Australia).

The inclusion criteria were (1) ≥18 years of age, (2) histologically confirmed diagnosis of AITL or PTCL-TFH, (3) availability of a pre-treatment AITL-containing tissue sample or bone marrow aspirate sample, (4) availability of at least one ctDNA sample subsequent to lymphoma diagnosis and (5) availability of sufficient clinical data to assign stages per Lugano criteria [36].

Following human research ethics committee approval, cases were identified using institutional databases and deidentified clinical and genomic data were subsequently collated and analysed.

Whole blood was uniformly collected in Cell-Free DNA BCT (Streck, La Vista, NE, USA) tubes from patients between July 2017 and January 2023 at multiple timepoints per institutional practice. These timepoints included pre-treatment, at time of interim PET/CT, at time of end-of-treatment PET/CT and during surveillance at approximately three-monthly intervals. ctDNA samples were considered paired with tissue samples or PET/CT if these occurred within one month.

Plasma was obtained from collected specimens by centrifugation at 1600× *g* for 10 min at room temperature, followed by transfer of plasma to a separate tube and further centrifugation at 16,000× *g* for 10 min. Extraction of ctDNA was completed using the automated QIAsymphony Circulating DNA kit (Qiagen, Hilden, Germany).

Pre-treatment formalin-fixed paraffin-embedded (FFPE) tissue samples and bone marrow aspirate samples were initially sequenced using a custom SureSelect (Agilent Technologies, Santa Clara, CA, USA) capture panel consisting of 73 genes recurrently mutated in haematological malignancy and run on a NextSeq 500 (Illumina, San Diego, CA, USA) with a minimum coverage of 150 reads. Variants were aligned and called using a customised bioinformatics pipeline. Called variants were then analysed using PathOS, an in-house variant caller [37]. Called variants with a variant allele frequency (VAF) of ≥5% were reviewed on the Integrative Genomics Viewer (IGV) to exclude artefacts [38]. Additionally, a focused review of *DNMT3A*, *IDH2*, *RHOA* and *TET2* called variants with a VAF of ≥2% was undertaken on IGV, given their particular relevance in AITL.

Due to superior assay sensitivity, tumour samples sequenced after 2021 and all ctDNA samples were tested on the QIAseq (Qiagen, Germantown, MD, USA) platform using a custom, single-primer-extension-based 56-gene panel utilising unique molecular index-based polymerase chain reaction (PCR) error correction—manufacturer catalogue number 333525.

Libraries were prepared using PCR through a sequence of fragmentation, end-repair and A-addition (incubated at 4 °C for 1 min, 32 °C for 24 min and 72 °C for 30 min and then held at 4 °C); ligation (incubated at 20 °C for 15 min and then held at 4 °C) and target enrichment (denaturation at 95 °C for 13 min, 98 °C for 2 min, then six cycles of 98 °C for 15 s followed by 65 °C for 15 min, then 72 °C for 5 min, and then held at 4 °C). Target-enriched libraries underwent universal PCR (denaturation at 95 °C for 13 min, 98 °C for 2 min, then 21 cycles of 98 °C for 15 s followed by 60 °C for 2 min, then 72 °C for 5 min and then held at 4 °C). Amplified PCR products were run on a NextSeq 500 (Illumina, USA) with 150 bp paired end reads with a minimum 500-read coverage to detect a VAF of ≥2%. A customised bioinformatics pipeline was utilised by incorporating CLC Genomic Workbench (Qiagen, USA) for unique molecular index calculation, alignment of reads and calling of variants against the hg19 human reference genome, followed by in-house processing to filter variants not meeting quality control metrics. Called variants were then analysed using PathOS. Additionally, a focused review of *DNMT3A*, *IDH2*, *RHOA* and *TET2* called variants with a VAF of ≥1% was undertaken on IGV, given their particular relevance in AITL.

Called variants were curated with reference to the Genome Aggregation Database (gnomAD, Broad Institute) and the Catalogue of Somatic Mutations in Cancer (COSMIC, Wellcome Sanger Institute) database to evaluate pathogenicity in the somatic context.

ctDNA somatic mutation burden was correlated with paired radiological response classified per Lugano criteria [36].

Exact binomial confidence intervals were calculated using R 4.4.2 (R Core Team, 2024).

## Figures and Tables

**Table 1:** Characteristics of tumour histology, staging and treatment of study patients.

Patient	Sex	Age at Diagnosis	Histology	Baseline Stage	Treatment	Interim PET/CT	End-of-Treatment PET/CT
1	M	73	AITL	Stage IV	CHOEP	CMR	CMR
2	M	72	AITL	Stage IV	CHOEP	CMR	CMR
3	F	57	AITL	Stage IV	HyperCVAD	CMR	CMR
4	M	77	AITL	Stage III	CHOEP	CMR	PD
5	F	67	AITL (relapsed)	Stage IV	Cyclosporin	NA	PD
6	F	75	AITL	Stage III	mini-CHOP	CMR	CMR
7	F	67	AITL	Stage III	CHOEP	PR	PR
8	F	51	PTCL-TFH	Stage II	CHOEP	CMR	CMR
9	M	75	AITL	Stage II	mini-R-CHOEP	CMR	CMR
10	F	56	AITL	Stage III	CHOP	CMR	CMR
11	F	59	AITL	Stage IV	HyperCVAD	CMR	CMR
12	F	76	PTCL-TFH	Stage IV	BV, cyclophosphamide, prednisolone	CMR	CMR

PET/CT: positron emission tomography/computed tomography; AITL: angioimmunoblastic T-cell lymphoma; BV: brentuximab vedotin; CHOEP: cyclophosphamide, doxorubicin, vincristine, etoposide, prednisolone CHOP: cyclophosphamide, doxorubicin, vincristine, prednisolone; CMR: complete metabolic response; HyperCVAD: cycles of cyclophosphamide, vincristine, doxorubicin, dexamethasone, alternating with cytarabine, methotrexate, methylprednisolone; PD: progressive disease; NA: not available; PR: partial response; PTCL-TFH: peripheral T-cell lymphoma of follicular helper cell phenotype.

**Table 2:** Somatic mutations detected in study patients.

Gene	P1	P2	P3	P4	P5 *	P6	P7	P8	P9	P10	P11 ^†^	P12 ^†^
*RHOA*	+	+	#	+		◊	◊	◊	◊		◊	+
*IDH2*	+	+		+		◊	◊	◊	◊		◊	
*TET2*	+@°	+	+#°	+	+	◊	◊	◊	◊		◊	+
*DNMT3A*	°	+	+	+	+	◊	◊				◊	+
*TP53*	°	°		°				°				
*NOTCH1*		@										
*KRAS*			°									
*ASXL1*				°								
*JAK2*									°			
*STAT5B*										◊		
*RUNX1*											°	
*STAT3*												+
Pre-treatment tissue	Y	Y	Y	Y	Y ^‡^	Y	Y	Y	Y	Y	Y	Y
Pre-treatment ctDNA	Y	Y	Y	Y	Y	N	N	N	N	N	N	Y
AITL relapse	N	Y	N ^§^	Y	Y ^‡^	Y	Y	N	N	N	N	N

* Bone marrow aspirate sample tested. ^†^ Pre-treatment tumour samples tested on the Qiaseq platform. ^‡^ Pre-treatment tumour sample was relapsed AITL. ^§^ Developed acute myeloid leukaemia post cytotoxic therapy. P: Patient. + Detected in both pre-treatment tumour sample and ctDNA sample. # Detected in pre-treatment tumour sample and not detected in pre-treatment ctDNA sample. ◊ Detected in pre-treatment tumour sample and pre-treatment ctDNA sample not available. @ Detected in pre-treatment ctDNA sample and not detected in pre-treatment tumour sample. ° Not detected in either pre-treatment tumour sample or pre-treatment ctDNA sample but detected in subsequent ctDNA sample.

## Data Availability

The original contributions presented in this study are included in the article. Further inquiries can be directed to the corresponding author.

## Supplementary Materials

The following supporting information can be downloaded at: https://www.mdpi.com/article/10.3390/ijms26146719/s1.
