# Supplementary material for: Circulating Tumour DNA Is a Biomarker of Response in Angioimmunoblastic T-Cell Lymphoma
Source: Int J Mol Sci. 2025 Jul 13;26(14):6719. doi: 10.3390/ijms26146719 (PMC12294795; doi:10.3390/ijms26146719)
Supplement: Supplementary file 1 [file ijms-26-06719-s001.zip › ijms-3716857-supplementary.pdf]

Supplementary Table S1

Patient	Gene	c. transcript	cDNA change	p. transcript	Amino acid change	In pre-treatment tissue (VAF%)	In pre-treatment ctDNA (VAF%)	In ctDNA at complete metabolic remission (VAF)%	In ctDNA during surveillance (VAF%)	In ctDNA following AITL relapse (VAF%)	In ctDNA following additional haematological malignancy relapse (VAF%)
1	*DNMT3A*	NM_022552.4	c.2644C>T	NP_072046.2	p.(R882C)	ND	ND	ND	8.2	NA	
1	*IDH2*	NM_002168.2	c.516G>T	NP_002159.2	p.(R172S)	15.3	10.9	ND	ND	NA	
1	*RHOA*	NM_001664.2	c.50G>T	NP_001655.1	p.(G17V)	11.4	12.0	ND	ND	NA	
1	*TET2*	NM_001127208.2	c.998del	NP_001120680.1	p.(P333Hfs*14)	ND	ND	ND	2.2	NA	
1	*TET2*	NM_001127208.2	c.1441C>T	NP_001120680.1	p.(Q481*)	16.1	13.2	ND	ND	NA	
1	*TET2*	NM_001127208.2	c.1630C>T	NP_001120680.1	p.(R544*)	ND	2.0	3.5	2.8	NA	
1	*TET2*	NM_001127208.2	c.4347_4354dup	NP_001120680.1	p.(R1452Lfs*9)	26.9	27.8	25.5	18.6	NA	
1	*TP53*	NM_000546.5	c.536A>G	NP_000537.3	p.(H179R)	ND	ND	ND	7.8	NA	
2	*DNMT3A*	NM_022552.4	c.958C>T	NP_072046.2	p.(R320*)	30.9	33.9	21.9	NA	41.0 (15 months after relapse)	
2	*IDH2*	NM_002168.2	c.515G>A	NP_002159.2	p.(R172K)	7.0	17.0	ND	NA	2.8 (15 months after relapse)	
2	*NOTCH1*	NM_017617.3	c.7207C>T	NP_060087.3	p.(Q2403*)	ND	8.3	ND	NA	ND	
2	*RHOA*	NM_001664.2	c.50G>T	NP_001655.1	p.(G17V)	13.4	14.5	ND	NA	1.7 (15 months after relapse)	
2	*TET2*	NM_001127208.2	c.3074_3089del	NP_001120680.1	p.(I1025Sfs*3)	14.0	37.9	15.2	NA	42.2 (15 months after relapse)	
2	*TET2*	NM_001127208.2	c.5615T>C	NP_001120680.1	p.(L1872P)	23.2	35.9	27.1	NA	42.4 (15 months after relapse)	
2	*TP53*	NM_000546.5	c.536A>G	NP_000537.3	p.(H179R)	ND	ND	ND	NA	2.2 (15 months after relapse)	
3	*DNMT3A*	NM_022552.4	c.1308C>G	NP_072046.2	p.(Y436*)	26.8	42.3	28.2	36.5	NA	43.9 (12 months after AML-pCT diagnosis)
3	*KRAS*	NM_033360.2	c.34G>T	NP_203524.1	p.(G12C)	ND	ND	ND	ND	NA	2.3 (12 months after AML-pCT diagnosis)
3	*RHOA*	NM_001664.2	c.50G>T	NP_001655.1	p.(G17V)	3.9	ND	ND	ND	NA	ND
3	*TET2*	NM_001127208.2	c.1102G>T	NP_001120680.1	p.(E368*)	ND	ND	ND	ND	NA	39.7 (12 months after AML-pCT diagnosis, also detected in bone marrow aspirate sample at time of AML-pCT)
3	*TET2*	NM_001127208.2	c.4587_4597del	NP_001120680.1	p.(P1530Afs*44)	20.2	34.6	25.3	27.3	NA	41.1 (12 months after AML-pCT diagnosis)
3	*TET2*	NM_001127208.2	c.4891dup	NP_001120680.1	p.(Y1631Lfs*30)	9.4	ND	ND	ND	NA	ND
4	*ASXL1*	NM_015338.5	c.3112A>G	NP_056153.2	p.(N1038D)	ND	ND	ND	ND	8.5	
4	*DNMT3A*	NM_022552.4	c.2645G>A	NP_072046.2	p.(R882H)	6.8	16.0	ND	2.0	13.4	
4	*IDH2*	NM_002168.2	c.516G>T	NP_002159.2	p.(R172S)	6.3	13.8	ND	1.3	11.0	
4	*RHOA*	NM_001664.2	c.50G>T	NP_001655.1	p.(G17V)	4.0	13.7	ND	ND	11.5	
4	*TET2*	NM_001127208.2	c.945del	NP_001120680.1	p.(Q317Rfs*30)	4.0	9.0	10.7	11.6	11.4	
4	*TET2*	NM_001127208.2	c.3805A>T	NP_001120680.1	p.(R1269*)	9.6	16.3	ND	1.8	12.5	
4	*TET2*	NM_001127208.2	c.4075C>T	NP_001120680.1	p.(R1359C)	9.9	17.4	ND	2.0	14.7	
4	*TP53*	NM_000546.5	c.824G>A	NP_000537.3	p.(C275Y)	ND	ND	ND	ND	12.2	
5*	*DNMT3A*	NM_022552.4	c.2578T>C	NP_072046.2	p.(W860R)	24†	22.6	NA (PET/CT Not available)	NA	NA	22.7 (at time of DLBCL diagnosis, also detected in DLBCL-containing tissue sample)
5*	*DNMT3A*	NM_022552.4	c.2645G>A	NP_072046.2	p.(R882H)	26.5†	16.9	NA (PET/CT Not available)	NA	NA	24.8 (at time of DLBCL diagnosis, also detected in DLBCL-containing tissue sample)
5*	*TET2*	NM_001127208.2	c.2200C>T	NP_001120680.1	p.(Q734*)	10.7†	9.7	NA (PET/CT Not available)	NA	NA	14.7 (at time of DLBCL diagnosis, also detected in DLBCL-containing tissue sample)
5*	*TET2*	NM_001127208.2	c.4393C>T	NP_001120680.1	p.(R1465*)	7.1†	4.3	NA (PET/CT Not available)	NA	NA	3.5 (at time of DLBCL diagnosis, also detected in DLBCL-containing tissue sample)
5*	*TET2*	NM_001127208.2	c.4664_4665del	NP_001120680.1	p.(E1555Vfs*22)	44.0†	40.7	NA (PET/CT Not available)	NA	NA	45.8 (at time of DLBCL diagnosis, also detected in DLBCL-containing tissue sample)
6	*DNMT3A*	NM_022552.4	c.919C>T	NP_072046.2	p.(P307S)	21.1	NA	NA (PET/CT Not available)	NA	31.7 (also detected in relapsed tissue sample)	
6	*IDH2*	NM_002168.2	c.515G>T	NP_002159.2	p.(R172M)	4.9	NA	NA (PET/CT Not available)	NA	1.4 (also detected in relapsed tissue sample)	
6	*RHOA*	NM_001664.2	c.50G>T	NP_001655.1	p.(G17V)	4.7	NA	NA (PET/CT Not available)	NA	1.2 (also detected in relapsed tissue sample)	
6	*TET2*	NM_001127208.2	c.961C>T	NP_001120680.1	p.(Q321*)	14.2	NA	NA (PET/CT Not available)	NA	31.0 (also detected in relapsed tissue sample)	
6	*TET2*	NM_001127208.2	c.2308C>T	NP_001120680.1	p.(Q770*)	8.9	NA	NA (PET/CT Not available)	NA	10.4 (also detected in relapsed tissue sample)	
6	*TET2*	NM_001127208.2	c.2539C>T	NP_001120680.1	p.(Q847*)	3.2	NA	NA (PET/CT Not available)	NA	12.6 (also detected in relapsed tissue sample)	
7	*DNMT3A*	NM_022552.4	c.1627G>A	NP_072046.2	p.(G543S)	4.9	NA	ND (remission after first relapse)	ND	15.9 (second relapse)	
7	*IDH2*	NM_002168.2	c.515G>T	NP_002159.2	p.(R172M)	2.8	NA	ND (remission after first relapse)	ND	17.3 (second relapse)	
7	*RHOA*	NM_001664.2	c.50G>T	NP_001655.1	p.(G17V)	2.5	NA	ND (remission after first relapse)	1.4 (15 months after first relapse)	16.6 (second relapse)	
7	*TET2*	NM_001127208.2	c.4761dup	NP_001120680.1	p.(I1588Yfs*26)	4.1	NA	ND (remission after first relapse)	ND	17.3 (second relapse)	
7	*TET2*	NM_001127208.2	c.5396del	NP_001120680.1	p.(K1799Rfs*21)	2.1	NA	ND (remission after first relapse)	3.2 (15 months after first relapse)	15.1 (second relapse)	
8	*IDH2*	NM_002168.2	c.516G>T	NP_002159.2	p.(R172S)	12.8	NA	ND	NA	NA	
8	*RHOA*	NM_001664.2	c.50G>T	NP_001655.1	p.(G17V)	18.9	NA	ND	NA	NA	
8	*TET2*	NM_001127208.2	c.3340dup	NP_001120680.1	p.(R1114Nfs*16)	22.9	NA	ND	NA	NA	
8	*TP53*	NM_000546.5	c.404G>A	NP_000537.3	p.(C135Y)	ND	NA	5.2	NA	NA	
9	*IDH2*	NM_002168.2	c.516G>T	NP_002159.2	p.(R172S)	3.8	NA	ND	ND	NA	
9	*JAK2*	NM_004972.3	c.1849G>T	NP_004963.1	p.(V617F)	ND	NA	12.6	11.0	NA	
9	*RHOA*	NM_001664.2	c.50G>T	NP_001655.1	p.(G17V)	3.5	NA	ND	ND	NA	
9	*TET2*	NM_001127208.2	c.3517A>T	NP_001120680.1	p.(K1173*)	17.4	NA	ND	ND	NA	
10	*STAT5B*	NM_012448.3	c.1994A>T	NP_036580.2	p.(Y665F)	2.0	NA	ND	NA	NA	
11	*DNMT3A*	NM_022552.4	c.1668G>T	NP_072046.2	p.(R556S)	34.3‡	NA	14.5	16.4	NA	
11	*IDH2*	NM_002168.2	c.516G>C	NP_002159.2	p.(R172S)	8.3‡	NA	ND	ND	NA	
11	*RHOA*	NM_001664.2	c.50G>T	NP_001655.1	p.(G17V)	7.5‡	NA	ND	ND	NA	
11	*RUNX1*	NM_001754.4	c.602G>A	NP_001745.2	p.(R201Q)	ND‡	NA	ND	2.4	NA	
11	*TET2*	NM_001127208.2	c.3076del	NP_001120680.1	p.(E1026Rfs*7)	32.3‡	NA	13.1	17.9	NA	
12	*DNMT3A*	NM_022552.4	c.2202C>G	NP_072046.2	p.(F734L)	2.9‡	8.2	20.3	NA	NA	
12	*RHOA*	NM_001664.2	c.50G>A	NP_001655.1	p.(G17E)	36.1‡	23.8	ND	NA	NA	
12	*RHOA*	NM_001664.2	c.76A>C	NP_001655.1	p.(S26R)	36‡	24.4	ND	NA	NA	
12	*STAT3*	NM_139276.2	c.1696G>A	NP_644805.1	p.(D566N)	9.6‡	16.4	ND	NA	NA	
12	*TET2*	NM_001127208.2	c.3927del	NP_001120680.1	p.(F1309Lfs*54)	38.1‡	27.8	ND	NA	NA	
12	*TET2*	NM_001127208.2	c.4045-1G>A	NP_001120680.1	p.?	41.7‡	26.6	ND	NA	NA	

ND - not detected

NA - not available

*Pre-treatment tumour sample was relapsed AITL

†Bone marrow aspirate sample tested

‡Pre-treatment tumour samples tested on the Qiaseq platform

IDH2/RHOA variants previously detectable within the ctDNA that are undetectable in complete metabolic response

DNMT3A/TET2 variant previously detected within the ctDNA that are detected in complete metabolic response

IDH2 or RHOA re-emergence in the ctDNA

Additional notes:

No corresponding remission PET/CT for patients 5 and 6
